# Epidemiological and therapeutic profiles of lung cancer patients in the Hokushin Region Japan: a retrospective hospital administrative database study

**DOI:** 10.1186/s12890-023-02610-5

**Published:** 2023-09-01

**Authors:** Takashi Kobayashi, Yoshikazu Nishino, Tomoya Takiguchi, Shintaro Kanda, Kengo Otsuki, Yuriko Tanaka, Yozo Nakazawa, Ken-ichi Ito, Ryuji Hayashi, Kazuo Yasumoto, Hidetaka Uramoto, Yasuo Hirono, Tomoe Makino, Mitsutoshi Nakada, Seiji Yano, Tomonobu Koizumi

**Affiliations:** 1 Department of Hematology and Medical Oncology, Shinshu University School of Medicine, 3-1-1 Asahi, Matsumoto, 390-8621 Nagano Japan; 2 Department of Epidemiology and Public Health, Kanazawa Medical University, Uchinada, Ishikawa Japan; 3 Department of Pediatrics, Shinshu University School of Medicine, Matsumoto, Japan; 4 Division of Breast and Endocrine Surgery, Department of Surgery, Shinshu University School of Medicine, Matsumoto, Japan; 5 Clinical Oncology, Toyama University Hospital, Toyama, Japan; 6 Department of Medical Oncology, Kanazawa Medical University, Uchinada, Ishikawa Japan; 7 Department of Thoracic Surgery, Kanazawa Medical University, Uchinada, Ishikawa Japan; 8 Cancer Care Promotion Center, University of Fukui Hospital, Fukui, Japan; 9 Division of Adult Nursing Practice, Ishikawa Prefectural Nursing University, Kahoku, Japan; 10 Department of Neurosurgery, Graduate School of Medical Science, Kanazawa University, Kanazawa, Japan; 11 Division of Medical Oncology, Cancer Research Institute, Kanazawa University, Kanazawa, Japan

**Keywords:** Cancer registration, Hospital-based cancer registry, Early-stage lung cancer, Lung cancer screening

## Abstract

**Objective:**

This study was performed to validate the epidemiology, initial treatment, and clinical practice of lung cancer patients in the Hokushin region, Japan.

**Methods:**

We retrospectively surveyed data of 5503 newly diagnosed and registered lung cancer patients in 22 principal hospital-based cancer registries in Hokushin region linked with health insurance claims data for registered patients between 2016 and 2017.

**Results:**

The patients consisted of 3677 (66.8%) men and 1826 (33.2%) women with a mean (range) age of 72.2 (27–103) years). Diagnoses were small cell lung cancer (*n* = 512, 9.4%), squamous cell carcinoma (*n* = 1083, 19.7%), and non-squamous non-small cell lung cancer (NSCLC; *n* = 3906, 70.9%). The population with stage I disease in Toyama prefecture (41.1%) was smaller than in the other three prefectures associated with reduced selection of initial surgical therapy and increased frequencies of stage IV disease (33.2%) and best supportive care (18.6%). Initial chemotherapy for stage IV non-squamous NSCLC consisted of tyrosine kinase inhibitors in 39.3% of cases for *EGFR* and 4% of cases for *ALK-*positive non-squamous NSCLC, followed by platinum compounds (25.9%) non-platinum compounds (12.9%), and immune checkpoint inhibitors (10.2%). Carboplatin was the commonly prescribed first-line cytotoxic chemotherapeutic agent (65.4% of patients under 75 years and in 96.7% of patients over 75 years).

**Conclusion:**

This study revealed real-world data on epidemiological and treatment status in lung cancer in four prefectures in Hokushin region, Japan. Simultaneous analysis of nationwide registry and insurance data could provide valuable insights for the development of lung cancer screening and medical treatment strategies. In addition, the comparative data analysis with other lesions or countries will be useful for evaluating the differences in clinical practice of cancer managements.

**Supplementary Information:**

The online version contains supplementary material available at 10.1186/s12890-023-02610-5.

## Introduction

Lung cancer is the leading cause of death from cancer worldwide, including Japan, with about 85% of cases presenting as non-small cell lung cancer (NSCLC) and the rest as small cell lung cancer (SCLC) [1–3]. The incidence of newly diagnosed lung cancer in Japan has been increasing, mainly in the elderly population [3–5]. It is necessary to reduce the number of lung cancer cases and deaths through a systematic and equitable implementation of evidence-based preventive strategies. Early discovery and/or screening for lung cancer are important to improve the mortality rate [6–9]. The Japanese government has promoted prefectural plans for cancer control programs and screening [8, 9]. Therefore, it is crucial to understand the region-specific demographics and backgrounds of patients with lung cancer. In addition, although each physician treated appropriately lung cancer patients according to the guideline [10], chemotherapy regimens, especially the use of platinum compounds, were depended on each physician’s choice or belonging to the hospital. Thus, chemotherapeutic approach and regimens might be different from the regions. Taken together, elucidation of trends in these parameters will be helpful to compose strategies to prevent and/or decrease the lung cancer mortality rate including management of lung cancer.

The Hokushin region of Japan is composed of the Hokuriku region (Fukui, Ishikawa, and Toyama prefectures) and Nagano prefecture (Supplemental Fig. 1), which have similarities in population age distribution, including high percentages of elderly residents aged ≥ 75 years. Based on a Cabinet Office annual report, the percentages of elderly residents aged 75 and above were much higher in Fukui (15.9%), Ishikawa (15.3%), Toyama (17.2%), and Nagano (17.5%) prefectures in comparison to the National data (14.9%) or for Tokyo (12.1%) (https://www.stat.go.jp/data/nihon/02.html). We established a large-scale database referred to as the “Hokushin Ganpro Database” to clarify the circumstances of cancer patients and reported data for rare tumors [11], the disabled population [12], and pediatric and adolescent and young adult populations [13] in the Hokushin region.

In the present study, we retrospectively surveyed data of patients with lung cancer using the Hokushin Ganpro Database and health care utilization data. We evaluated the epidemiology, stage distribution, and initial treatments in patients with lung cancer. In addition, we examined the initial chemotherapeutic approach and frequency in use of platinum compounds for stage IV lung cancer in the Hokushin region to compare real-world practice in patients with metastatic lung cancer.

## Materials and methods

### The Hokushin Ganpro Database and health care utilization data

“Hokushin Ganpro” is the name of the educational program implemented by the Ministry of Education, Culture, Sports, Science and Technology of Japan (https://gan-pro.net/) to enable improved cancer treatment by training highly skilled health care professionals via cooperation among universities in the Hokushin region (Kanazawa University, Kanazawa Medical University, Shinshu University, The University of Toyama, The University of Fukui, and Ishikawa Prefectural Nursing University).

The Hokushin Ganpro Database 2 is a regional cancer database created as a project of the Hokushin Ganpro and built from the hospital-based cancer registry (HBCR)-linked health care utilization data, i.e., the so-called Diagnosis Procedure Combination (DPC) survey data. Collection of DPC data was performed as part of a governmental survey to assess the effects of introduction of the diagnostic procedure combination-based payment system. The survey data included information equivalent to fee-for-service insurance claims covering all billable health services (e.g., diagnostic tests, imaging workup, procedures, treatments, and prescribed drugs) for both inpatients and outpatients. These data were linked to the HBCR data of each patient in the participating hospitals. The Hokushin region has 28 Ministry of Health, Labor and Welfare-designated cancer care hospitals, in which approximately 35 000 people are registered and diagnosed with cancer every year. Twenty of these hospitals and two other non- designated cancer care hospitals participated in Hokushin Ganpro data set 2 (Supplemental Table 1).

The definition of malignancy in the present study corresponds to behavioral codes 2 or 3 in the International Classification of Disease for Oncology, 3rd edition (ICD-O-3). All targeted lung cancers newly encountered at hospitals between January 1, 2016, and December 31, 2017, were registered. The interval of DPC data corresponding to HBCR in the Hokushin Ganpro Database was selected from October 1, 2015, to July 31, 2017. We analyzed the patients in Class of Cases 20 and 30. These are coded as 20 (diagnosed and treated initially in the registering hospital) and 30 (diagnosed in another hospital and treated initially in the registering hospital), respectively. The histological types and the codes for lung cancer included SCLC (codes 80,413, 80,453), adenocarcinoma (codes 81,402, 81,403, 81,413, 82,003, 82,113, 82,303, 82,503, 82,523, 82,533, 82,543, 82,553, 82,603, 82,633, 82,653, 83,103, 84,803, 84,813, 85,503, 85,513), squamous cell carcinoma (codes 80,523, 80,702, 80,703, 80,713, 80,723, 807,433, 80,823, 80,833), neuroendocrine tumors (codes 80,133, 82,403, 82,463, 82,493), large cell carcinoma (codes 80,123), adenosquamous cell carcinoma (code 85,603), and others (codes 80,003, 80,013, 80,102, 80,103, 80,203, 80,223, 80,313, 80,323, 80,333, 80,463, 84,303, 85,743, 89,723).

As of 2015, the Center for Cancer Control and Information Services of the National Cancer Center collected and managed HBCR data from 427 designated cancer care hospitals [14]. The national data in Japan were drawn from the National Cancer Registry [2]. This study was approved by the Institutional Review Board of Shinshu University School of Medicine (No.5054) and Kanazawa University (No.2750-4). Institutional Review Board approval for collecting the anonymized data and creating the database was also obtained from each participating facility. The need for informed consent was waived by the Institutional Review Boards of Shinshu University School of Medicine (No.5054) and Kanazawa University (No.2750-4) due to the retrospective nature of the study and the use of anonymized data. The data set was used with permission from the Data Utilization Committee of Hokushin Ganpro Database Project (Institutional Review Board of Kanazawa University: No.2750-4). These materials and methods described above were similar with those in our other published studies [15, 16]. Pearson’s χ^2^ test was used to compare the ages and usage of platinum agents between the < 75 years and ≥ 75 years age groups.

## Results

### Patients

Table 1 shows the number of lung cancer patients included in this study. A total of 5503 patients with a mean (range) age of 72.2 (27–103) years were registered in the Hokushin area. These patients consisted of 3677 men with a mean (range) age of 72.6 (29 – 101) years and 1826 women with a mean (range) age of 71.6 (27 – 103) years. There were no significant differences in male/female ratio or mean age among the four prefectures in the Hokushin area, and the male/female ratios were similar to the national average.

**Table 1:** Numbers and age of lung cancer (NSCLC and SCLC) cases in the Hokushin region, 2016–2017

	Toyama		Ishikawa		Fukui		Nagano		Hokushin region		National data
	Cases (%)	Mean age ± SD (range)	Cases (%)	Mean age ± SD (range)	Cases (%)	Mean age ± SD (range)	Cases (%)	Mean age ± SD (range)	Cases (%)	Mean age ± SD (range)	Cases (%)
Male	1111 (68.8)	73.1 ± 9.8 (29–101)	956 (66.2)	71.6 ± 8.7 (38–95)	443 (70.0)	72.3 ± 9.5 (32–92)	1167 (66.4)	72.9 ± 9.1 (33–99)	3677 (66.8)	72.6 ± 9.3 (29–101)	90,103 (67.0)
Female	503 (31.2)	73.9 ± 10.5 (38–103)	488 (33.8)	69.5 ± 10.5 (27–98)	190 (30.0)	71.9 ± 10.8 (39–100)	645 (35.6)	71.3 ± 11.3 (30–96)	1826 (33.2)	71.6 ± 11.0 (27–103)	44,387 (33.0)
Total	1614	73.3 ± 10.0 (29–103)	1444	70.9 ± 9.4 (27–98)	633	72.2 ± 9.9 (32–100)	1812	72.3 ± 10.0 (30–99)	5503	72.2 ± 99 (29–103)	134,490

### Stage distribution

**Fig. 1:**
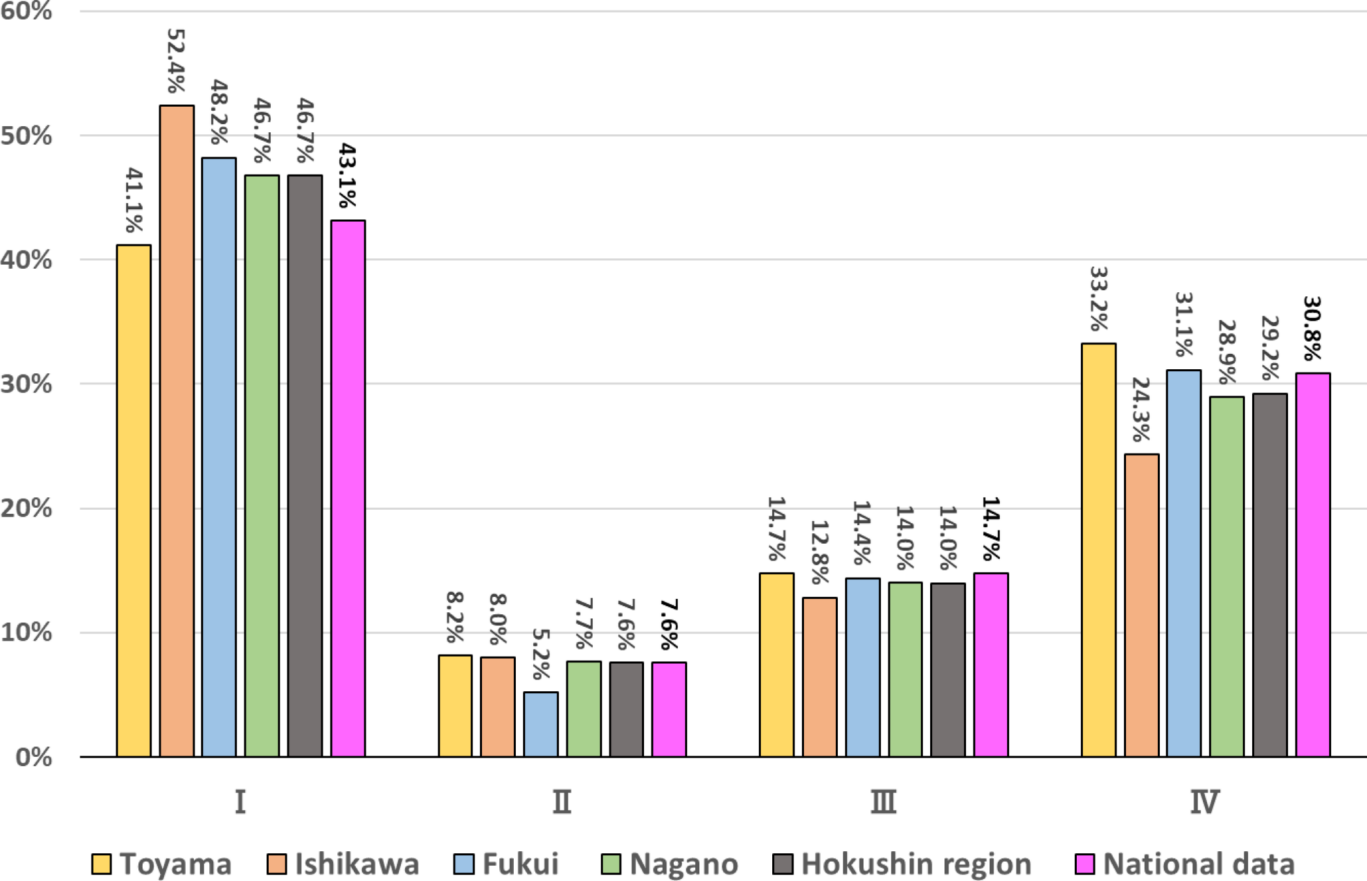
Stage distribution of lung cancer at first diagnosis ;comparison between Hokushin region (Toyama, Ishikawa, Fukui and Nagano prefecture) and all Japan (National data)

The stage distributions of lung cancer in the Hokushin region and in each individual prefecture are shown in Fig. 1. The mean stage I detection rate was slightly higher, while the mean stage IV detection rate was slightly lower in Hokushin region than in the Japan national data (46.7% vs. 43.1% and 29.2% vs. 30.8%, respectively). There were differences in the frequencies of stage I and IV lung cancer among the four prefectures in the Hokushin region. The frequency of stage I (41.1%) was lower, while that of stage IV was higher (33.2%) in Toyama prefecture than the other three prefectures.

### Initial therapy

**Fig. 2:**
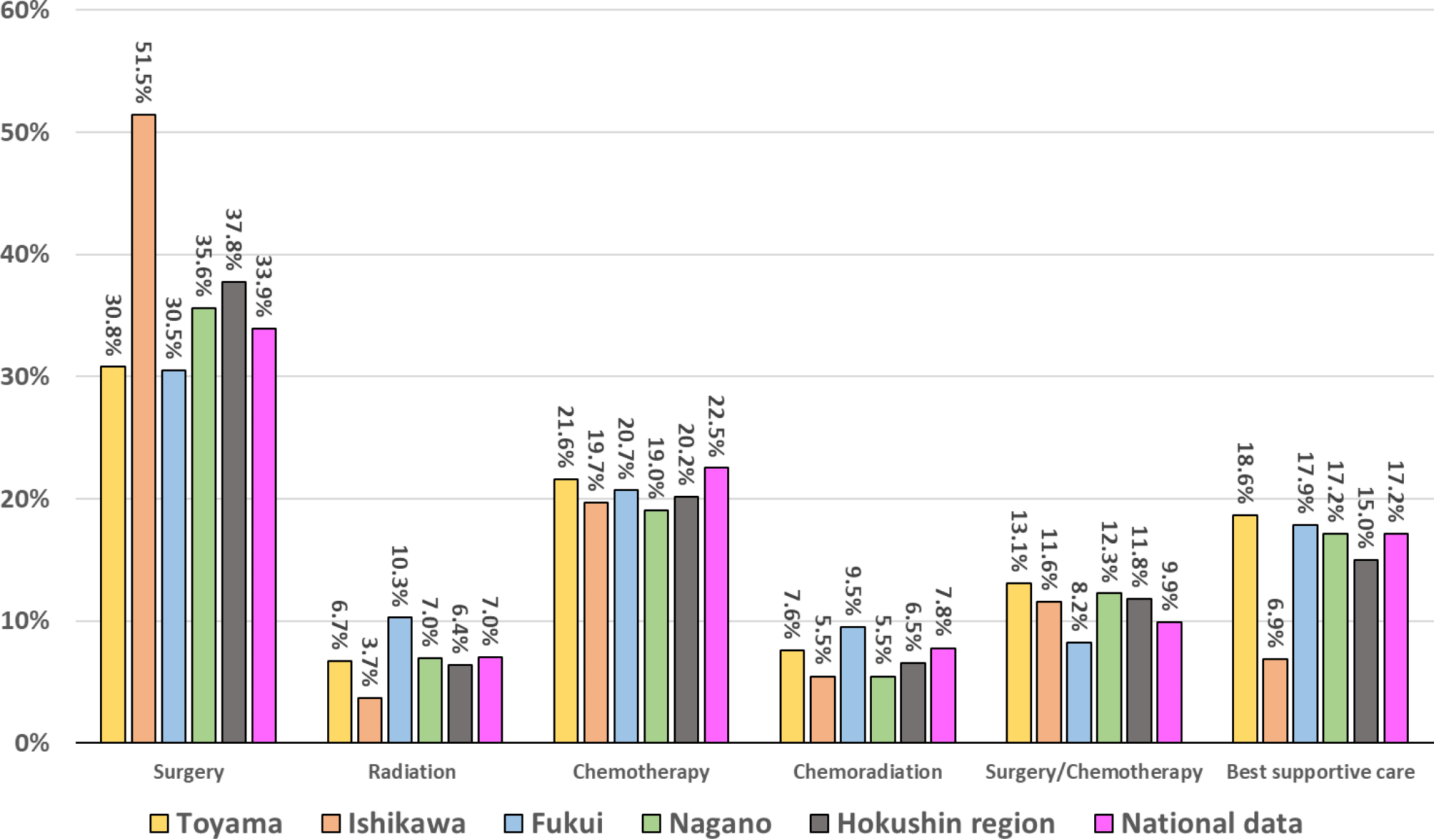
Proportion of initial therapies for lung cancer; comparison between Hokushin region (Toyama, Ishikawa, Fukui and Nagano prefecture) and all Japan (National data)

The results of analyses of initial therapies for lung cancer in the Hokushin region are shown in Fig. 2. The mean frequency of surgery in the Hokushin region was 37.8%, which was slightly higher than in the Japan national data (31.9%). The frequency in Ishikawa prefecture was highest among the four prefectures in the Hokushin region, and was related to the highest frequency of stage I lung cancer in this prefecture (Fig. 1). Fukui prefecture had relatively high rates of selection of radiation alone and chemoradiotherapy. There were high incidences of best supportive care (BSC) among patients with lung cancer in both the Japan national data and Hokushin area, although the rate in Hokushin region was slightly lower (15.0% vs. 17.2%, respectively). Stage IV patients accounted for 51.7% of those in the BSC group in Hokushin region, and the frequency was highest in Toyama prefecture, which may have been related to the highest rate of stage IV disease in this prefecture. Furthermore, the mean (range) age was significantly higher in the BSC group than the treatment group in the Hokushin region [80.1 (38–103) years vs. 71.1 (27–94) years, respectively, *p* < 0.0001].

### Initial agents as first-line chemotherapy

Table 2 shows the chemotherapeutic drugs used for initial treatment of non-squamous NSCLCs. In the Hokushin region, epidermal growth factor receptor (EGFR)-tyrosine kinase inhibitors (TKIs) were used in 39.3% of cases, anaplastic lymphoma kinase (ALK) inhibitors in 4.0%, and immune checkpoint inhibitors in 10.2%. Platinum combined therapy was used in 38.8% of cases and non-platinum single-drug therapy was used in 7.7%. In the Hokushin region, Fukui prefecture showed a slightly lower rate of EGFR-TKI use and higher rate of platinum combined therapy. Non-platinum single-drug therapy was used at a slightly higher rate in Ishikawa prefecture. We analyzed the frequency of use of cisplatin and carboplatin in patients with lung cancer in Hokushin region, and the data are summarized in Table 3. In patients aged < 75 years in the Hokushin region, carboplatin was used in 65.4% of cases and cisplatin in 34.6%. There were marked differences in rates of therapy using these drugs among individual prefectures. The rates of carboplatin use in Toyama prefecture and Ishikawa prefecture were 75.2% and 73.5%, respectively. Cisplatin was used in 46.3% of cases in Fukui prefecture and 44.2% of cases in Nagano prefecture. Carboplatin was used in most patients aged ≥ 75 years in the Hokushin region.

**Table 2:** Agents for initial treatment of non-squamous stage IV NSCLCs

Agents	Toyama	Ishikawa	Fukui	Nagano	Hokushin region
	Cases (%)	Cases (%)	Cases (%)	Cases (%)	Cases (%)
EGFR-TKIs	67 (44.1%)	60 (42.0%)	18 (27.7%)	60 (40.0%)	205 (39.3%)
ALK inhibitor	5 (3.1%)	9 (6.3%)	2 (3.1%)	5 (3.3%)	21 (4.0%)
ICIs	19 (11.7%)	15 (10.5%)	5 (7.7%)	14 (9.3%)	53 (10.2%)
Platinum compound	61 (37.4%)	39 (27.3%)	37 (56.9%)	65 (43.3%)	202 (38.8%)
Non-platinum	11 (6.7%)	20 (14.0%)	3 (4.6%)	6 (4.0%)	40 (7.7%)
Total	163	143	65	150	521

**Table 3:** Numbers of patients treated with platinum agents for lung cancer (NSCLC and SCLC)

Age	Platainum	Toyama	Ishikawa	Fukui	Nagano	Hokushin region
		Cases (%)	Cases (%)	Cases (%)	Cases (%)	Cases (%)
< 75	Carboplatin	91 (75.2%)	75 (73.5%)	43 (53.8%)	63 (55.8%)	272 (65.4%)
	Cisplatin	30 (24.8%)	27 (26.5%)	37 (46.3%)	50 (44.2%)	144 (34.6%)
≥ 75	Carboplatin	48 (96.0%)	28 (96.6%)	11 (100%)	30 (96.8%)	117 (96.7%)
	Cisplatin	2 (4.0%) *	1 (3.4%) *	0 (0%) *	1 (3.2%) *	4 (3.3%) *
Total		171	131	91	144	537

* p < 0.001, statistical significant compared with < 75 years

## Discussion

Here, we examined the lung cancer status of 5503 newly diagnosed cases from 2016 to 2017 in the Hokushin region based on the Hokushin Ganpro data set 2, which consisted of HBCRs and DPCs. The distributions of histological type, sex, and age were similar between prefectures, although we found several differences in the stage distribution and initial therapy among prefectures.

The mean stage I detection rate in the Hokushin region was slightly higher than the Japan national data. However, the frequency of stage I disease in Toyama prefecture was lower, while that of stage IV disease was higher than in the other three prefectures and the national data. In general, detecting lung cancer in the early stage and the shift to early stage of lung cancer at discovery could improve lung cancer survival [6, 7]. Our comparative analysis between prefectures in the Hokushin region provided useful information for each prefecture to address countermeasures against cancer. Our epidemiological analysis showed that innovative methods and/or widespread adoption of cancer screening are required for the early detection of lung cancer.

Unfortunately, there were high incidences of BSC among patients with lung cancer in the Hokushin area. Older age and advanced lung cancer stage were closely correlated with BSC without any specific cancer treatment options in the present study. Our findings regarding BSC patients with lung cancer are clinically important for understanding the circumstances around lung cancer in this region. With the aging of society in Hokushin region (https://www.stat.go.jp/data/nihon/02.html), cancer control should be considered in elderly patients to help administrators in this region.

The 2016 Guideline for Treatment of Lung Cancer of The Japan Lung Cancer Society recommends testing patients with non-squamous NSCLC for multiple biomarkers, including *EGFR* gene mutation, *ALK* fusion, and programmed cell death 1 (PD-L1) expression [10]. TKIs (grade A evidence) were recommended as first-line treatments in patients with stage IV NSCLC with tumors harboring *EGFR* or *ALK* mutations. We analyzed initial chemotherapy agents in patients with non-squamous NSCLC treated with chemotherapy using the DPC survey data. In our clinical practice in Hokushin area, 39.3% and 4% of patients with non-squamous NSCLC were treated with EGFR- and ALK-TKIs, respectively. These frequencies were consistent with a retrospective observational study conducted in 2017 (the BRAVE study; 34.7% in *EGFR*, 6.9% in *ALK*) regarding the choice of first-line chemotherapy in cases of advanced and metastatic NSCLC [17]. In contrast, 10.2% of patients in the Hokushin region received anti-PD-1 antibody, which was slightly lower than the rate of 22.2% in the BRAVE study [17]. As first-line pembrolizumab, an anti PD-L1 antibody, became available in December 2016 in Japan, the lower usage of anti-PD-L1 antibody in the present study was due to an inadequate interval of data collection. First-line chemotherapy for lung cancer has advanced with use of PD-1 inhibitors combined with cytotoxic chemotherapy and/or anti-cytotoxic T-lymphocyte antigen 4 (CTLA-4) antibody [18]. Serial and further analyses of real-world data will be important for decision-making regarding treatment in routine clinical practice.

Platinum doublet therapy is the standard chemotherapy in patients with *EGFR/ALK*-negative/unknown status NSCLC and non–platinum-based chemotherapy is recommended for use in elderly patients (> 75 years) [14]. In a clinical trial of first-line platinum-based chemotherapy versus chemotherapy plus pembrolizumab for NSCLC without *EGFR* or *ALK* mutations, carboplatin and cisplatin were used in 72.3% and 27.8% of cases, respectively [19]. Several studies using DPC survey data revealed first-line treatment patterns in patients with lung cancer [19, 20]. Combined analysis using HBCR and DPC data showed that carboplatin was commonly prescribed in all age groups, and only 28.4% received cisplatin doublet chemotherapy [21]. Although the pattern of platinum usage in the Hokushin region was similar, the rates were quite different between the four prefectures. Therefore, these real-world data regarding clinical practice and the comparison among prefectures based on the cancer registry and DPCs provide valuable insight for the selection of chemotherapy.

Despite the valuable findings, our study had several limitations. The results of testing for biological markers (*EGFR* mutation, *ALK* rearrangement, and PD-L1 expression) were lacking. Second, the Hokushin Ganpro Database does not necessarily contain all HBCR data in the Hokushin region. Therefore, care should be taken when comparing the numbers and stage distribution between prefectures. Third, the interval of data sampling in Hokushin Ganpro data set 2 was only 2 years, which may have been insufficient to evaluate the epidemiological trends in lung cancer. Nevertheless, we applied DPC survey data corresponding to each registered case in the Hokushin Ganpro data set in the present study. We could analyze the details of these cases, especially with regard to treatment regimens, and our findings represent further informative data of real-world practice in lung cancer in the Hokushin region. Further serial studies examining the cancer registry data in this region are required.

## Conclusion

We described the epidemiological and clinical situation of lung cancer in four prefectures in Japan using Hokushin Ganpro data set 2. The cancer registry system, including health care utilization data, is a valuable resource for evaluating disease trends and treatment in these areas, and provides valuable insights for the development of lung cancer screening and medical treatment strategies. Further serial analyses of these real-world data will improve cancer prevention and management in this region. In addition, comparative data analysis with other lesions or countries will be important for cancer control.

### Electronic supplementary material

Below is the link to the electronic supplementary material.

Supplementary Material 1

Supplementary Material 2

## Data Availability

All data generated or analyzed during this study are included in this published article and its supplementary information.

## Abbreviations

ALK: Anaplastic lymphoma kinase
BSC: Best supportive care
DPC: Diagnosis Procedure Combination
EGFR: Epidermal growth factor receptor
HBCR: Hospital-based cancer registry
ICD-O-3: International Classification of Disease for Oncology, 3rd edition
NSCLC: Non-small cell lung cancer
PD-L1: Programmed cell death 1
CTLA-4: Cytotoxic T-lymphocyte antigen 4
SCLC: Small cell lung cancer
