# Supplementary material for: Epidemiological and therapeutic profiles of lung cancer patients in the Hokushin Region Japan: a retrospective hospital administrative database study
Source: BMC Pulm Med. 2023 Sep 1;23:322. doi: 10.1186/s12890-023-02610-5 (PMC10472700; doi:10.1186/s12890-023-02610-5)
Supplement: Supplementary file 1 — Supplementary Material 1 [file 12890_2023_2610_MOESM1_ESM.docx]

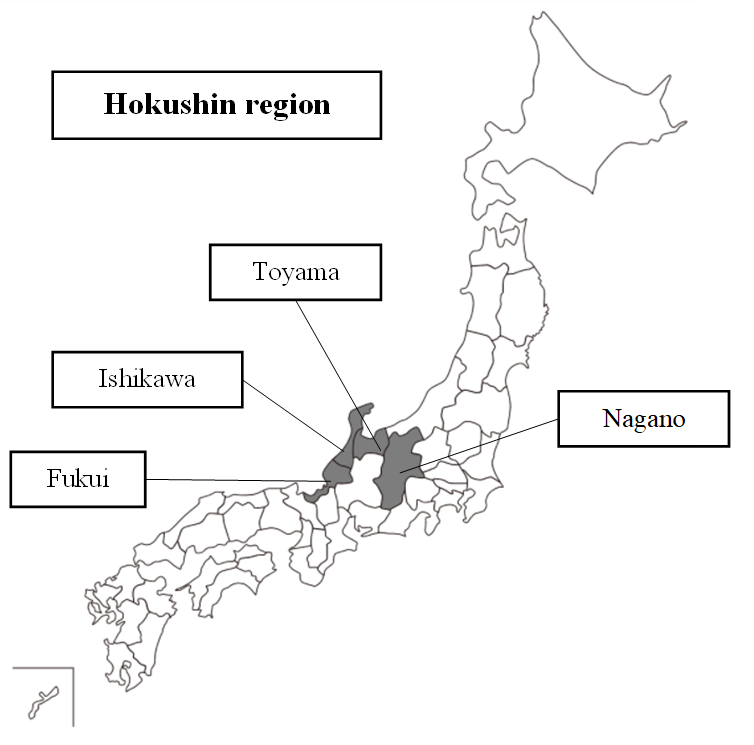


Supplementary Fig. 1 The Hokushin region is composed of the Fukui, Ishikawa, Toyama, and Nagano prefectures, which have commonalities, such as the population’s age distribution and snowy climates during the winter.
