# Supplementary material for: Epidemiological and therapeutic profiles of lung cancer patients in the Hokushin Region Japan: a retrospective hospital administrative database study
Source: BMC Pulm Med. 2023 Sep 1;23:322. doi: 10.1186/s12890-023-02610-5 (PMC10472700; doi:10.1186/s12890-023-02610-5)
Supplement: Supplementary file 2 — Supplementary Material 2 [file 12890_2023_2610_MOESM2_ESM.docx]

Supplementary Table 1: Lists of participating hospitals in the present study.

| Fukui prefecture | Fukui University Hospital | Ishikawa prefecture | Kanazawa University Hospital |
| --- | --- | --- | --- |
|  | Fukui Prefectural Hospital |  | Kanazawa Medical University Hospital |
|  | National Hospital Organization Tsuruga Medical Center |  | Ishikawa Prefectural Central Hospital |
| Toyama prefecture | Toyama University Hospital |  | National Hospital Organization Kanazawa Medical Center |
|  | Toyama City Hospital | Nagano prefecture | Shinshu University Hospital |
|  | Kurobe City Hospital |  | Nagano Municipal Hospital |
|  | Toyama Rosai Hospital |  | Nagano Red Cross Hospital |
|  | JA Toyama Kouseiren Takaoka Hospital |  | Suwa Red Cross Hospital |
|  | Takaoka City Hospital |  | Nagano Children’s Hospital |
|  | Tonami General Hospital, |  | Aizawa Hospital |
|  | Toyama Prefectural Central Hospital |  | Saku Central Hospital Advanced Care Center |
